# Inhibitor of Apoptosis Proteins in Pediatric Leukemia: Molecular Pathways and Novel Approaches to Therapy

**DOI:** 10.3389/fonc.2014.00003

**Published:** 2014-01-27

**Authors:** Simone Fulda

**Affiliations:** ^1^Institute for Experimental Cancer Research in Pediatrics, Goethe-University, Frankfurt, Germany

**Keywords:** IAP proteins, Smac, apoptosis, leukemia, lymphoma

## Abstract

Inhibitor of Apoptosis (IAP) proteins are a family of proteins with antiapoptotic functions that contribute to the evasion of apoptosis, a form of programed cell death. IAP proteins are expressed at high levels in a variety of human cancers including childhood acute leukemia. This elevated expression has been associated with unfavorable prognosis and poor outcome. Therefore, IAP proteins are currently exploited as therapeutic targets for cancer drug discovery. Consequently, small-molecule inhibitors or antisense oligonucleotides directed against IAP proteins have been developed over the last years. Indeed, IAP antagonists proved to exhibit *in vitro* and *in vivo* antitumor activities against childhood pediatric leukemia in several preclinical studies. Thus, targeting IAP proteins represents a promising molecular targeted strategy to overcome apoptosis resistance in childhood leukemia, which warrants further exploitation.

## Introduction

Cancer cells have typically acquired the ability to evade apoptosis, a form of programed cell death (1). In addition, the evasion of apoptotic cell death contributes to treatment resistance, since most anticancer therapies act by engaging this intrinsic program of cell death in cancer cells (2). For example, antiapoptotic proteins can block apoptosis (2). Inhibitor of apoptosis (IAP) proteins are a family of endogenously expressed proteins that block signal transduction to apoptosis (3). Of note, expression and/or function of IAP proteins are altered in various cancers including childhood leukemia. Therefore, IAP proteins are considered as promising targets for drug discovery. Several key discoveries made over the last decade have provided insights into the various functions of IAP proteins and their regulation in human cancers. In addition, major advances have been achieved in the development of therapeutic strategies to antagonize IAP proteins. This review focuses on the role of IAP proteins and their therapeutic targeting in pediatric leukemia.

## Apoptotic Signal Transduction

There are two key signaling pathways to apoptosis, namely, the death receptor (extrinsic) pathway and the mitochondrial (intrinsic) pathway (4). Signal transduction via the death receptor pathway starts with binding of death receptor ligands of the tumor necrosis factor (TNF) receptor superfamily to their related cell surface receptors, for example TNFα to TNF receptor, CD95 ligand to CD95, and TNF-related apoptosis-inducing ligand (TRAIL) to TRAIL receptors. This leads to activation of caspase-8 at the death-inducing signaling complex (DISC). Once activated, caspase-8 either directly cleaves effector caspase-3 or, alternatively, cleaves Bid and thereby engages the mitochondrial apoptosis pathway (4). The mitochondrial (intrinsic) pathway involves the release of mitochondrial intermembrane proteins into the cytosol, including cytochrome *c* and second mitochondria-derived activator of caspases (Smac) (5). Smac facilitates caspase-3, -7, and -9 activation by binding and neutralizing IAP proteins (5). By comparison, cytochrome *c* supports the assembly of the apoptosome complex, leading to caspase-9 and -3 activation (5).

## IAP Proteins: Structure and Function

The family of IAP proteins comprises eight human analogs (3). Among them, cellular IAP1 (cIAP1), cIAP2, X-linked inhibitor of apoptosis (XIAP), melanoma-IAP (ML-IAP), and survivin have been most extensively characterized (3). All IAP proteins contain at least one baculoviral IAP repeat (BIR) domain of 70–80 amino acids. Additional domains include the Really Interesting New Gene (RING) domain, an E3 ubiquitin ligase responsible for ubiquitination and proteasomal degradation of substrates (6), and the Caspase-Activating and Recruitment Domain (CARD), a protein–protein interaction domain for oligomerization with other CARD domain-containing proteins. XIAP is considered as the IAP family member with the strongest antiapoptotic activities (7) and blocks apoptosis by binding to and inhibiting activation of caspase-3, -7, and -9 (3).

## IAP Proteins in Pediatric Leukemia

Expression levels of IAP proteins are elevated in a variety of human malignancies including pediatric leukemia, which may be caused by genetic events as well as by transcriptional or posttranscriptional mechanisms. It is important to note that aberrant expression of IAP proteins was described to correlate with adverse patients’ outcome, suggesting that IAP proteins bear a prognostic impact.

### XIAP, cIAP1, and cIAP2 in pediatric leukemia

The prognostic significance of XIAP has been studied in childhood acute myeloid leukemia (AML). XIAP has been associated with adverse prognosis in pediatric leukemia by independent studies showing a correlation of high mRNA and protein expression levels of XIAP and several unfavorable prognostic parameters including high-risk groups for cytogenetics, immature morphology, poor treatment response to induction chemotherapy, and reduced relapse-free survival (8, 9). These findings indicate that XIAP represents an indicator of poor prognosis in pediatric AML. Also in childhood T-cell acute lymphoblastic leukemia (ALL), elevated expression levels of XIAP protein turned out to be an unfavorable prognostic factor, as there was a correlation between high XIAP protein expression and poor prednisone response in T-cell ALL (10). Of note, this correlation was specifically found for XIAP protein rather than for XIAP mRNA expression, suggesting that XIAP levels are controlled by posttranslational or posttranscriptional mechanisms (10). Consistently, XIAP belongs to the set of factors that harbor an internal ribosomal entry site (IRES), which initiates translation even under conditions of intracellular stress when protein synthesis is normally shut down (11).

### ML-IAP in pediatric leukemia

In childhood ALL, a large study comprising the analysis of 222 patients showed that high expression levels of ML-IAP mRNA correlated with a favorable rather than an unfavorable prognosis (12). In addition, patients with higher XIAP expression exhibited a better bone marrow response upon induction chemotherapy at day 7 compared to patients with lower ML-IAP levels (12). Also, ML-IAP turned out to be an independent favorable prognostic factor in multivariate analysis for relapse-free survival of children with ALL (12). These findings are particularly remarkable, since ML-IAP gene expression has been linked to poor prognosis in adult acute leukemia (13). While the reasons for this differential impact of ML-IAP in childhood and adult acute leukemia have not been identified, ML-IAP has been described to exhibit both anti- and proapoptotic activities. On one hand, ML-IAP binds to Smac and also promotes degradation of caspases via its ubiquitin E3 ligase activity, thereby inhibiting apoptosis (14), and on the other hand, the truncated form of ML-IAP (i.e., tML-IAP) that is generated upon its cleavage by caspases has been reported to promote apoptosis (15).

### Survivin in pediatric leukemia

Overexpression of survivin was detected in two-thirds of precursor B-cell ALL samples in contrast to negligible expression levels in non-malignant hematopoietic cells (16). Higher survivin expression was associated with a higher risk of disease relapse or death and also turned out to be a significant prognostic marker for 3-year relapse-free, event-free, and overall survival (16). Analysis of survivin splice variants in pediatric precursor B-cell ALL showed an association between lower survivin-2B expression and affiliation to the high-risk group (17). Furthermore, high expression levels of survivin were reported to correlate with poor overall survival in childhood *de novo* AML (9). A recent analysis of survivin mRNA levels and survivin transcript splice variants on diagnostic bone marrow samples from children with *de novo* AML showed that high survivin-2B/ΔEx2 ratios were associated with refractory disease and inferior survival in childhood AML (18).

## Targeting IAP Proteins for the Treatment of Childhood Leukemia

Since expression levels of IAP proteins were found to be elevated in pediatric acute leukemia and since IAP proteins are known as potent inhibitors of cell death, they are currently viewed as potential targets for therapeutic intervention. For example, small-molecule inhibitors that mimic the N-terminal part of the endogenous IAP antagonist Smac were designed. In addition to monovalent IAP antagonists, also bivalent compounds with higher potency were designed that are composed of two monovalent motifs connected via a central chemical link.

### IAP antagonists in combination with TRAIL or CD95 ligand

In childhood ALL, IAP antagonists at subtoxic concentrations were shown to cooperate with the death receptor ligand TRAIL to trigger apoptosis in a synergistic manner (19). The specificity of the IAP antagonist-mediated sensitization toward TRAIL was supported by data showing that a structurally related control compound failed to sensitize ALL cells to TRAIL-induced cell death (19). The synergistic induction of apoptosis by IAP antagonists and TRAIL was accompanied by enhanced activation of caspases, loss of mitochondrial membrane potential, and cytochrome *c* release (19). Of note, XIAP antagonists even bypassed the Bcl-2-imposed block to TRAIL-mediated apoptosis (19). Furthermore, IAP antagonists triggered apoptosis in primary leukemic blasts derived from children with ALL (19). *In vivo*, IAP antagonists succeeded to reduce the leukemic burden in an NOD/SCID mouse model of childhood ALL (19). Besides the death receptor ligand TRAIL, IAP antagonists were found to cooperate with agonistic anti-CD95 antibodies or a hexameric form of the CD95 ligand to trigger apoptosis in pediatric ALL cells (20).

### IAP antagonists in combination with TNFα

Furthermore, IAP antagonists have also been reported to act together with TNFα, another death receptor ligand, in order to synergistically trigger cell death in leukemia cells (21–23). Recently, Smac mimetic in conjunction with TNFα was shown to engage necroptosis as an alternative form of programed cell death in apoptosis-resistant ALL cells that were genetically deficient for Fas-associated death domain (FADD) or caspase-8 (22). Receptor-Interacting Protein (RIP)1 was critically required for this type of cell death, which lacked typical features of apoptotic cell death such as caspase activation or DNA fragmentation (22). These studies demonstrate that IAP antagonists can potentiate TNFα-stimulated cell death by promoting either apoptosis or necroptosis, depending on the cellular context.

### IAP antagonists in combination with anticancer agents

In addition to death receptor agonists, it was also reported that IAP antagonists act in concert with various anticancer drugs, including AraC, Gemcitabine, Cyclophosphamide, Doxorubicin, Etoposide, Vincristine, and Taxol, to trigger apoptosis in ALL cells in a synergistic manner (24). Of note, IAP antagonists failed to sensitize normal peripheral blood lymphocytes for Cytarabine-induced apoptosis, similar to their failure to enhance the sensitivity to TRAIL- or CD95-mediated apoptosis (19, 20, 24). Also, IAP antagonists did not enhance the cytotoxic effects of Cytarabine on normal human hematopoietic progenitor cells or mesenchymal stromal cells (24). The chemosensitization of ALL cells by IAP antagonists was found to critically depend on the serine/threonine kinase RIP1 that was required to form a cytosolic cell death complex containing RIP1/FADD/caspase-8, thereby driving caspase-8 activation (24). Furthermore, IAP inhibition using the Smac mimetic LBW242 significantly increased prednisone-induced apoptosis in a precursor B-cell ALL cell line (10).

### Survivin inhibition

Knockdown of survivin by either short-hairpin RNA (shRNA) or a locked antisense oligonucleotide was reported to induce apoptosis in leukemia cell lines and also potentiated the chemotherapeutic antileukemic effects (25–27). In addition, a survivin-locked antisense oligonucleotide resulted in a significant inhibition of tumor progression in a mouse primary xenograft model of relapse ALL (25). Based on preclinical studies suggesting that targeting endogenous levels of survivin mRNA may augment the response to chemotherapy, a phase 1 study combining a survivin mRNA antagonist, EZN-3042, with re-induction chemotherapy was recently conducted in childhood relapsed ALL (28). However, the study has been prematurely terminated, since this regimen turned out to be too toxic at a dose that was required for downregulation of survivin expression and since the clinical development of EZN-3042 was discontinued (28).

## Conclusion

IAP proteins are promising novel targets for molecular targeted therapy in childhood leukemia. Agents antagonizing IAP proteins, including small-molecule inhibitors and antisense oligonucleotides, have been shown to trigger apoptotic and non-apoptotic cell death alone or in combination in preclinical studies in pediatric leukemia. Currently, IAP antagonists are under evaluation in early clinical trials in adult leukemia. Thus, IAP-targeting treatment strategies warrant further clinical investigation in childhood leukemia.

## Conflict of Interest Statement

The author declares that the research was conducted in the absence of any commercial or financial relationships that could be construed as a potential conflict of interest.
